# Second harmonic generation analysis of early Achilles tendinosis in response to *in vivo *mechanical loading

**DOI:** 10.1186/1471-2474-12-26

**Published:** 2011-01-26

**Authors:** Thomas Abraham, Gloria Fong, Alex Scott

**Affiliations:** 1 Centre for Hip Health, Vancouver Coastal Health Research Institute, University of British Columbia, Vancouver, Canada; 2 Department of Physical Therapy, University of British Columbia, Vancouver, Canada; 3 James Hogg Research Centre-St Paul's Hospital, Vancouver, Canada

## Abstract

**Background:**

Tenocytes have been implicated in the development of tendinosis, a chronic condition commonly seen in musculoskeletal overuse syndromes. However, the relation between abnormal tenocyte morphology and early changes in the fibrillar collagen matrix has not been closely examined *in vivo*. Second harmonic generation (SHG) microscopy is a recently developed technique which allows examination of fibrillar collagen structures with a high degree of specificity and resolution. The goal of this study was to examine the potential utility of SHG and multiphoton excitation fluorescence (MPEF) microscopy in understanding the relation between tenocytes and their surrounding collagenous matrix in early tendon overuse lesions.

**Methods:**

Histological preparations of tendon were prepared from adult male Sprague-Dawley rats subjected to an Achilles tendon loading protocol for 12 weeks (Rat-A-PED), or from sedentary age-matched cage controls. Second harmonic generation and multiphoton excitation fluorescence were performed simultaneously on these tissue sections in at least three different areas.

**Results:**

SHG microscopy revealed an association between abnormal tenocyte morphology and morphological changes in the fibrillar collagen matrix of mechanically loaded Achilles tendons. Collagen density and organization was significantly reduced in focal micro-regions of mechanically loaded tendons. These pathological changes occurred specifically in association with altered tenocyte morphology. Normal tendons displayed a regular distribution of fibre bundles, and the average size of these bundles as determined by Gaussian analysis was 0.47 μm ± 0.02. In comparison, fibre bundle measures from tendon regions in the vicinity of abnormal tenocytes could not be quantified due to a reduction in their regularity of distribution and orientation.

**Conclusions:**

SHG microscopy allowed high resolution detection of focal tendon abnormalities affecting the fibrillar collagen matrix. With ongoing repetitive loading, these tenocyte-associated focal collagen defects could predispose to the progression of overuse pathology.

## Background

Multiphoton and associated microscopy methods have been widely used for imaging dynamic interactions in cells and tissues with submicron resolution [1-3]. Among these methods, the second harmonic generation (SHG) imaging method shares many of the features of multi photon excitation fluorescence (MPEF) microscopy, including identical equipment requirements and intrinsic capability of generating 3D images. Highly ordered ECM macromolecules such as the fibril-forming collagens produce SHG signal without the need for an exogenous label. The SHG signals derived from collagen macromolecules are strongly depend on the collagen density, where SHG signal intensity (*I_SHG_*) scales as *I_SHG _*≈ *N*^2 ^Here *N *is the effective number of radiating molecules. Likewise, isotropic macromolecules generate MPEF signal due to their endogenous fluorescence. Multiphoton excitation fluorescence intensity (*I_MPEF_*) emitted by these molecules undergoing multiphoton excitation can be expressed as, *I_MPEF _*≈ *N*. While SHG signal intensity scales as a square of number of collagen molecules, the MPEF intensity scales linearly with the number of other endogenously fluorescent molecules. Since MPEF and SHG involve different principles and contrast mechanisms, SHG and MPEF can be captured simultaneously to visualize structural changes of biological macromolecules. Particularly, SHG signals originating from the collagen structures can be used to generate high resolution images of individual collagen fibre bundles and quantify collagen density in a given tissue. Hence, SHG provides the potential to investigate precise spatial relations between collagen organization and underlying tenocyte biology.

Tendinosis is a histological entitity characterized by collagen remodeling and angiofibroblastic hyperplasia. Tendons which rupture often demonstrate evidence of pre-existing tendinosis [5]. However, initiating events in the development of tendinosis have not been closely examined. There is increasing recognition of the need to detect early signs of tendon injury so that appropriate prevention strategies can be implemented, as the pathology is frequently career- or activity-limiting [6]. The imaging modalities currently in use to investigate the structural reorganization of collagen in tendon tissues are primarily based on conventional light microscopy. Histochemical or immunolabeling procedures are considered at best semi-quantitative, since these methods are highly sensitive to the accessibility of the epitope in antigens recognized by the antibodies. In addition, bright field or fluorescent images of immunolabeled tissue samples are always convolved with significant amount of background, which make the quantification further problematic. Higher resolution transmission electron microscopy, while able to visualize collagen alterations with greater sensitivity, imposes other limitations including the need to dissect samples into smaller pieces which can obscure the spatial patterns in which abnormalities occur [7,8].

Research into the early pathogenesis of tendinosis has suggested that tenocytes may initiate degradation and remodelling of the load-bearing collagen matrix in response to mechanical loading. This model runs counter to a traditional "wear and tear" hypothesis of collagen microtrauma [9]. Dudhia et al. demonstrated that in response to cyclic physiologic loading, tendon explants which contained viable tenocytes experienced a significant loss of mechanical integrity, whereas under the same loading conditions, explants without viable tenocytes did not experience a loss of load-bearing function [10]. Cyclic loading has also been shown to increase the expression of matrix metalloproteinases by tenocytes, which would support a model of tenocyte-driven changes of potential relevance to understanding early pathological change [11-13]. A cross-sectional clinical studies identified abnormal tenocyte morphology as a potential early feature of tendinosis [14].

The goal of the current study was to determine whether SHG microscopy could be used to examine a potential association between abnormal tenocyte morphology and local reductions of collagen density in a relevant *in vivo *model of mechanical overload. We hypothesized that collagen density and fibrillar organization would be significantly reduced in highly localized areas associated with abnormal tenocyte morphology.

## Methods

### Animals

Twelve male Sprague-Dawley rats aged 4 months, weight 448.7 ± 10 grams were obtained locally and housed singly in standard cage conditions. Following a 48 hour acclimatization period, rats were divided into controls (n = 6, standard cage care), or runners (n = 6, standard cage care plus treadmill running), providing 12 tendons per group. Ethic approval for the study was obtained from the UBC animal care committee (A07-0274).

### Tendon mechanical loading

Runners were subjected to a previously published mechanical loading protocol (Rat-A-PED) using a dedicated rat treadmill to induce Achilles tendinosis (Exer 3/6, Columbus Instruments, Columbus, OH) [15]. Rats were acclimatized to the treadmill by gradually increasing their exposure over a 2 week period. Subsequently, the treadmill was adjusted to a 10 degree uphill grade. Following the acclimatization period, rats ran for 1 hour / day at 1 km / hr. After 12 weeks of exercise or sedentary cage activity, rats rested for 72 hours.

### Tissue sampling and processing

Rats were euthanized with CO_2_, and whole bilateral Achilles tendons were removed with the muscle still attached, oriented longitudinally in Tissue Tek embedding medium (Tissue Tek, Sakura Finetek, Torrance, CA), then frozen in isopentane chilled in liquid nitrogen. Tendons were cryosectioned onto charged glass slides at 5 micron thickness and processed for H&E (general morphology), Alcian blue / fast nuclear red (stains sulphated glycosaminoglycans and cell nuclei), or Picrosirius red (enhances birefringence of collagen when viewed under polarized light).

### Multiphoton and second harmonic generation microscopy

The microscope system used in our present experiments is the same as described previously [1]. Specifically, the laser used for SHG as well as the MPEF was a mode-locked femto-second Ti:Sapphire Tsunami (Spectra-Physics, Mountain View, CA) synchronously pumped by a Millenia Xs J (Spectra-Physics) diode-pumped solid-state laser capable of delivering up to 10 W pumping power at 532 nm. The power attenuated laser was directed to a Leica AOBS RS scan head (4000 Hz) coupled with Leica upright microscope system (Heidelberg, Germany). The laser beam was focused on the specimen through Lecia water immersion objectives. The water immersion objectives used and their lateral resolution (*R_xy_*) values follow: 20X/0.7 NA (*R_xy_*≈0.50 μm); 63X/1.2 NA (*R_xy_*≈0.292 μm). Upon entering the Leica microscope system, the laser beam was directed to the scanning mirrors, then through a 670 nm long pass dichroic mirror (RSP 670, Leica) and subsequently focused on the specimens through the objective lens. The backscattered emission from the sample was collected through the objective lens. Leica Confocal Software TCS SP2 was used for the image acquisition. Non-descanned detectors and spectral scanning mode both in the reflection geometry were used for capturing the MPEF images as well as for the spectral signal characterizations respectively. In the non-descanned PMT detectors (R6357, Hamamatsu, Shizuoka, Japan), a 700 nm short pass filter (E700SP, Chroma Technology, USA) was used to prevent the scattered IR laser radiation from reaching the detector and a 455 long pass dichroic beam splitter (455 DCXRU, Chroma Technology, USA) was used to separate SHG signal from the MPEF signal. SHG signal in forward direction was captured using a non-descanned detector in the transmission geometry equipped with a 440/20 nm band pass filter (MP 440/20, Chroma Technology) and high NA condenser. All SHG and MPEF spectral data were generated using the de-scanned PMT detector (R6357, Hamamatsu) located inside the scan head where the emission signals were delivered through the AOBS detection system with the maximum confocal pinhole setting at 600 μm via the spectral dispersion prism. The width of the slits in front of each PMT could be software adjusted such that each PMT could detect spectral regions spanning from a 5 nm bandwidth up to the overall spectral capacity of the system (400-800 nm). With this instrument configuration, a series of individual images were collected using a narrow detection window with a width of ~5 nm, and each image detected at this specific emission wavelength band (i.e. ~ 5 nm) provided a data point in the spectral graph. The gain and the offset of the PMTs were adjusted for optimized detection using the color gradient to avoid pixel intensity saturation and background. Images (8 bit) acquired at slow scan speed i.e. 10 sec per 512 X 512 pixels.

### Statistical analysis

Lateral line profiles of collagen fibers were generated using Leica comprehensive image processing software from 2D spectrally clean SHG images. The sizes of the collagen fibers and their size distribution were plotted by fitting the lateral line profiles to a Gaussian curve using Origin Lab Software.

A Mann-Whitney U test was used to detect differences in collagen density and blood flow. Values are expressed as means ± SE. Results were considered statistically significant if α was less than 0.05.

## Results

### SHG and MPEF images of normal rat Achilles tendon

**Figure 1:**
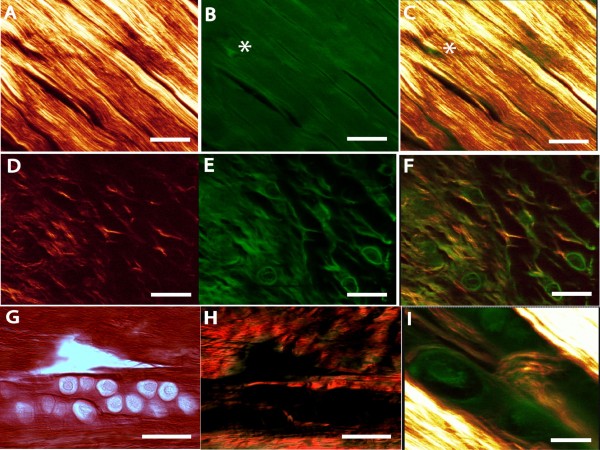
**Multiphoton and SHG signals originating from tendon histological preparations**. MPEF images show general tissue morphology of tendon and associated structures, while spectrally clean SHG images specifically reveal collagen present in the same region. Representative SHG (A,D), MPEF (B,E) or combined images (C,F,I) obtained from standard histological thin tissue section are shown. In normal healthy tendon (A-C), SHG demonstrates tightly bundled, longitudinally oriented collagen fibres. Tenocytes (B, C, asterisk) are inconspicuous due to their sparse cytoplasm. In early tendinosis tendon, collagen surrounding abnormal tenocytes (D-F) demonstrates a disturbed organization, and the average SHG signal is greatly reduced (c.f. panel A). The tenocytes in tendinosis tendon demonstrate much more prominent cytoplasm and a rounded, as opposed to spindle-shaped, morphology (E). Picrosirius red-stained tendon from tendinosis tendon (G, brightfield; H, polarized light) is shown as a comparison. Tendinosis regions demonstrate a loss of birefringence (H). In comparison, SHG signal reveals the presence of abnormal pericellular fibrillar collagen structures not visualized with polarized light. Scale bars represent 25 μm for A-H, and 10 μm for I.

In order to determine the microregional properties of collagen surrounding abnormal tenocytes in mechanically loaded tendon, we assessed standard histological preparations using multiphoton and second harmonic generation (SHG) microscopy. Initially, normal tendon was analyzed. Representative images originating from spectrally clean SHG signal originating from collagen (1A) and the MPEF signal originating from endogenously fluorescent tissue components (Figure 1B) are shown. A broad range of infrared laser excitation wavelengths (from 800 to 900 nm), with a scan interval of 10 nm were employed to detect the SHG signal. The emission spectrum obtained from the wavelength scan revealed a strong SHG signal manifested by a narrow peak at 440 nm, which is exactly half of the excitation wavelength (i.e. 880 nm). Similar infrared laser excitation wavelength (i.e. 880 nm) was used to generate the MPEF images. There was a strong SHG signal originating from the collagen-rich tendon-proper region. In contrast, muscle fibres generated no signal and intramuscular connective tissue only a very faint signal. Likewise, paratendinous structures including vessels and their surrounding extracellular matrix and adipose tissue, emitted virtually undetectable signals. This is in keeping with the known distribution of fibrillar collagens within these tissues [16]. Thus, when examining musculotendinous anatomical preparations, the SHG signal was extremely specific for load-bearing collagen-rich tendinous structures.

### Collagen fibrillar organization and tenocyte abnormality

After confirming the specificity of collagenous structures visualized with SHG in normal tendons, we next examined the collagen organization in sedentary and mechanically loaded tendons. All Achilles tendons analyzed from animals subjected to the mechanical loading protocol demonstrated microregions of abnormal tenocyte morphology, typified by the appearance of tenocytes with prominent cytoplasm and rounded morphology (Figure 1D-F). These morphological features were absent in normal rat Achilles tendons, consistent with the original description of this experimental model [15]. Abnormally appearing tenocytes in tendons from rats subjected to mechanical loading demonstrated increased Alcian Blue staining both intra- and pericellularly (Additional file 1, Figure S1), and decreased birefringence of Picrosirius Red stained sections examined with polarized light (Figure 1H). Collagen fibres in the vicinity of abnormal tenocyte morphology displayed a reduced SHG signal level and a distinctive pericellular organization suggestive of localized remodelling activity (Figure 1D-F, H). This abnormal appearance of fibrillar collagen occurred in highly localized microregions surrounding abnormal-appearing tenocytes. These microregions were distributed throughout the midsubstance of the Achilles tendon. In contrast, normal tendon displayed regularly spaced, tightly packed fibrillar collagen, with slender spaces occupied by elongated tenocytes.

### Quantitative analysis of SHG signal in tendinopathy

**Figure 2:**
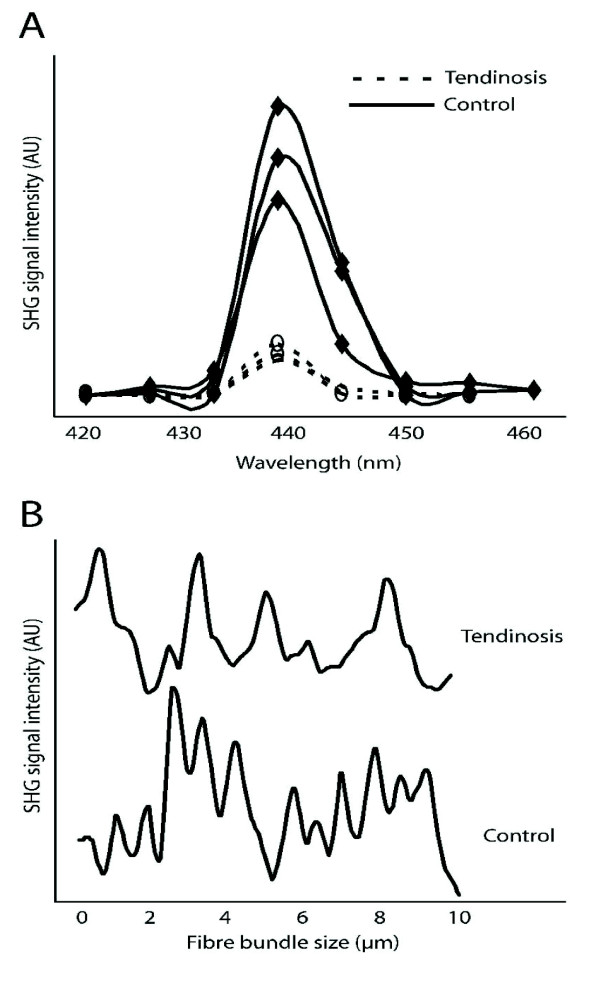
**Analysis of SHG signal intensity in rat Achilles tendon from collagen surrounding normal or abnormal tenocytes**. (A) Three representative spectral scans from a single normal (solid line) and a single tendinosis tendon (dashed line) are shown. The SHG signal arising from the tendon proper region peaked consistently at 440 nm as expected. (B) When SHG signal intensity was plotted as a function of position along a line drawn perpendicular to the collagen fibre bundles, the fibre bundle size could be estimated with sub-micron resolution in normal tendon, but not in tendinosis tendons.

SHG signals emitted from the Achilles tendon proper region in normal tendon vs tendinosis lesions were next characterized by spectral analysis. The wavelength scans of both normal tendon from sedentary rats, and of tendon surrounding abnormal tenocytes from rats subjected to the mechanical loading protocol, showed a highly specific SHG signal manifested by a narrow peak at 440 nm (Figure 2). The resolution of collagenous structures visualized with SHG could be estimated by plotting SHG signal intensity vs distance (along a digital line placed perpendicular to the direction of fibres). Normal tendons displayed a regular distribution of fibre bundles (Figure 2B, lower plot), and the average size of these bundles as determined by Gaussian analysis was 0.47 μm ± 0.02. In comparison, fibre bundle measures from tendon regions in the vicinity of abnormal tenocytes displayed a less regular distribution and orientation, which made analysis of fibre bundle size not feasible (Figure 2B, upper plot).

We then applied a noise removal filter to SHG images to define the boundary between foreground and background, and the lower threshold in the histogram was set to 10% of the highest pixel intensity value. The total SHG signal intensity values thus generated were normalized by the cropped collagen area (μm^2^) to yield a mean collagen density value. The collagen density from micro-regions corresponding to abnormal tenocytes in rats subjected to the overuse protocol was 729 ± 90.3 AU, compared to the value of 1135 ± 60.1 in normal tendon (p < 0.05).

## Discussion

Second harmonic generation (SHG) resulted in high resolution, spectrally clean signals originating from fibrillar collagen. The collagen matrix surrounding abnormal tenocytes demonstrated an abnormal organization and a reduced density compared to sedentary cage controls. This reduction in SHG signal occurred specifically in association with the appearance of abnormal tencoytes in the tendons subjected to mechanical loading. These results provide support to an emerging model of tendinosis where degradation and/or remodelling of the load-bearing collagen matrix in response to exercise is mediated by the local activity of tenocytes, rather than being due to a passive accumulation of fatigue damage [10,14].

Glazebrook et al. recently reported *increased *collagen staining using this overuse tendinopathy model based on assessment of H&E preparations [15]. However, we are confident that the *reduced *SHG signal observed in the current study accurately reflects a loss of collagen density in the tendinosis samples. Indeed, H&E evaluation of tendinosis samples may be misleading as tendinosis lesions can be highly eosinophilic, but we have found these eosinophilic regions to generate very weak or absent SHG signals indicating that they may comprise provisional matrix with a higher component of non-collagenous elements. We are therefore able here to refine and further validate the description of early pathology which occurs in this model.

In the current study, SHG was used to estimate the collagen fibre bundle diameter with submicron resolution in normal tendons, but not in tendinosis samples. The longitudinal alignment of collagen fibres was reduced in the tendinosis tendons, in association with a reduction of SHG signal intensity. The technique of measuring fibre bundle diameter relied on the fibres demonstrating an orderly, longitudinal orientation, which was disrupted in areas of tendinosis.

## Conclusions

Second harmonic generation analysis of mechanically loaded tendon provides the ability to examine collagen organization in standard histological preparations, providing insight into mechanisms of early load-induced tendon changes. Tenocyte responses to mechanical loading *in vivo *are accompanied by highly localized foci of reduced collagen density which may predispose to the progression of overuse tendinopathy.

## Competing interests

The authors declare that they have no competing interests.

## Authors' contributions

AS and TA conceived of the study. AS supervised the tendon mechanical loading protocol and carried out the imaging and histological examination. GF assisted with imaging analysis. TA and AS conducted the image analysis. TA designed and oversaw the SHG and MPEF microscopy. All authors read and approved of the final manuscript.

## Authors' information

Alex Scott is a physical therapist engaged in full time research and teaching. Gloria Fong is a Research Assistant with a BSc in Medical and Laboratory Sciences. Thomas Abraham is a specialist in optical physics engaged in full time research.

## Pre-publication history

The pre-publication history for this paper can be accessed here:

http://www.biomedcentral.com/1471-2474/12/26/prepub

## Supplementary Material

Additional file 1 **Figure 1S Glycosaminoglycan distribution in mechanically loaded and sedentary tendon**. Alcian blue staining in tendinosis (A) and normal healthy (B) tendon. Tendinosis tendon demonstrates increased sulphated glycosaminoglycan intra- and peri-cellularly, particularly in chains of abnormal-appearing tenocytes. Mast cells also stain blue with this technique in the paratendon regions (not shown). 20× objective lens. Click here for file
