# Supplementary figures and images for: Second harmonic generation analysis of early Achilles tendinosis in response to in vivo mechanical loading
Source: BMC Musculoskelet Disord. 2011 Jan 26;12:26. doi: 10.1186/1471-2474-12-26 (PMC3045393; doi:10.1186/1471-2474-12-26)

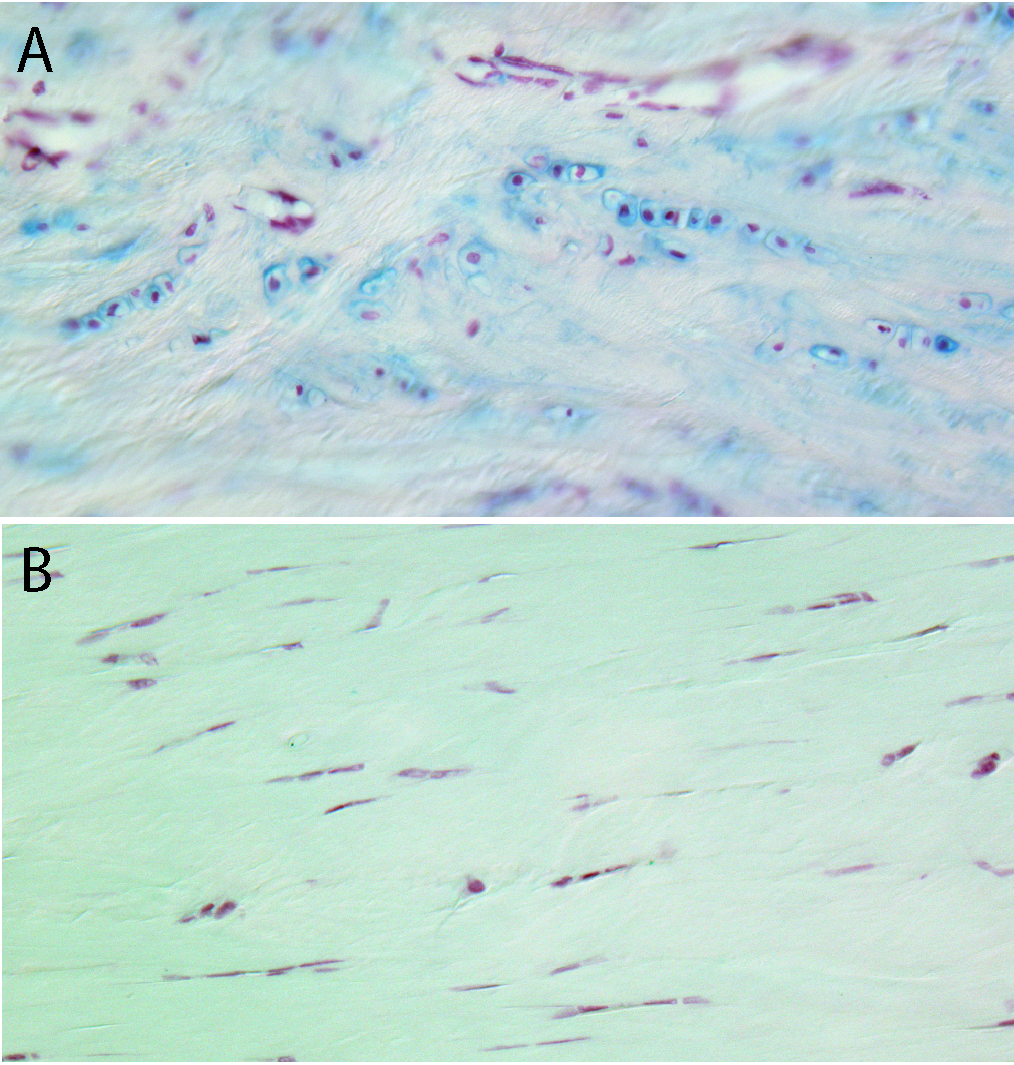

Supplement: Additional file 1 — Figure 1S Glycosaminoglycan distribution in mechanically loaded and sedentary tendon. Alcian blue staining in tendinosis (A) and normal healthy (B) tendon. Tendinosis tendon demonstrates increased sulphated glycosaminoglycan intra- and peri-cellularly, particularly in chains of abnormal-appearing tenocytes. Mast cells also stain blue with this technique in the paratendon regions (not shown). 20× objective lens. [file 1471-2474-12-26-S1.TIFF]
